# NADPH-mediated seedless in situ formation of gold or gold-platinum nanoparticles for the enzymatic determination of atropine

**DOI:** 10.1007/s00604-025-06964-x

**Published:** 2025-02-04

**Authors:** Mario Domínguez, Susana de Marcos, Javier Galbán

**Affiliations:** 1 Analytical Chemistry Department, University of Zaragoza, 50009 Saragossa, Spain; 2 Instituto de Nanociencia y Materiales de Aragón (INMA), CSIC-Universidad de Zaragoza, 50009 Saragossa, Spain

**Keywords:** Gold nanoparticles, Gold-platinum nanoparticles, Tropinone reductase, Tropane alkaloid, NADH

## Abstract

The without-seeds in situ formation of gold nanoparticles from NADPH and its application to the colorimetric determination of atropine (a tropane alkaloid) in cereals is reported. The method is based on a chemical hydrolysis, followed by an enzymatic oxidation by NADP catalyzed by tropinone reductase in the presence of Au(III) or Au(III)/Pt(II). During this reaction, the formed NADPH reduces the metal ion precursor to AuNPs (or AuPtNPs) and the absorption due to the plasmon band (550 nm or 575 nm) is measured. The method (AuPtNPs) allows the determination of the analyte in the concentration range 0.025 to 0.09 mM with an RSD of 3% (*n* = 5) and is applied to its determination in spiked buckwheat samples using the standard addition method, with 96.9 ± 2.0% recovery. It is also demonstrated that NAD(P)H and NADH have different kinetics for AuNP generation, which could be used to discriminate between these two cofactors.

**Graphical Abstract:**

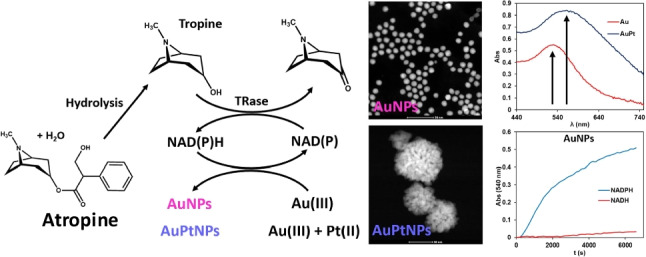

**Supplementary Information:**

The online version contains supplementary material available at 10.1007/s00604-025-06964-x.

## Introduction

Atropine is, along with scopolamine, the most important and abundant tropane alkaloid (TA). It has an anticholinergic effect, so in controlled doses it has pharmacological properties (i.e. anti-sickness, anti-shock or bronchodilator) [1] and is also used as an eye dilator for medical examinations, but in medium/high doses it has devastating effects on human health. The problem is that atropine can be found in significant amounts in foods such as tea, aniseed and especially in products made from various types of cereal. This is because atropine is a secondary metabolite produced in plants of several families (such as *Brassicaceae*, *Solanaceae*, and some others) whose seeds contaminate other plants such as linseed, soya, sorghum, millet or sunflower [2, 3].

There seems to be no agreement between [4] on the lethal dose of atropine in the human organism but, although this varies among different studies, it can be said to be around 100 mg for adults and 10 mg (or less) for children. This low lethal dose has encouraged various organizations to establish a maximum allowable concentration of atropine in some foods. In 2016, the European Union (EU) set a maximum concentration of 1 mg/kg of atropine in cereals for children [5]. Later, in 2021, the EU will set a limit for other foods such as tea (25 mg /kg), aniseed (50 mg /kg) or non-transformed cereals (between 5 and 25 mg/kg) [6]. To have accurate information on these concentrations, the European Food Safety Authority (EFSA) has sponsored several studies to identify the levels of atropine and other TAs in different foods in Europe [7]. As a result, many samples revealed concentrations up to 400 mg/kg, other studies have also reported high concentrations compared to the recommended limits [3, 8] and even extremely high concentrations have been found in some samples (more than 3000 mg/kg and even a sample containing 25,000 mg/kg has been reported [9]).

The analytical methods used in laboratories for measuring atropine (and other TAs) usually involve chromatographic techniques [10], especially HPLC, with the complexity of sample handling depending on the detector used. A very commonly reported setup is HPLC–MS/MS [7], in which most of the sample treatment involves a first step of extraction with polar solvents and different additional operations such as filtration through a 30KD column [11, 12] or a more recent method based on QuEChERS using SPE [13]. These methods have given satisfactory results and are used in laboratories. For rapid, continuous and even in situ control, ELISA assays have been developed [14, 15] and commercialized [16]. These methods give good results in terms of sensitivity and selectivity, but as they are irreversible and very expensive, they are not universally used for rapid and frequent control of food samples.

**Fig. 1:**
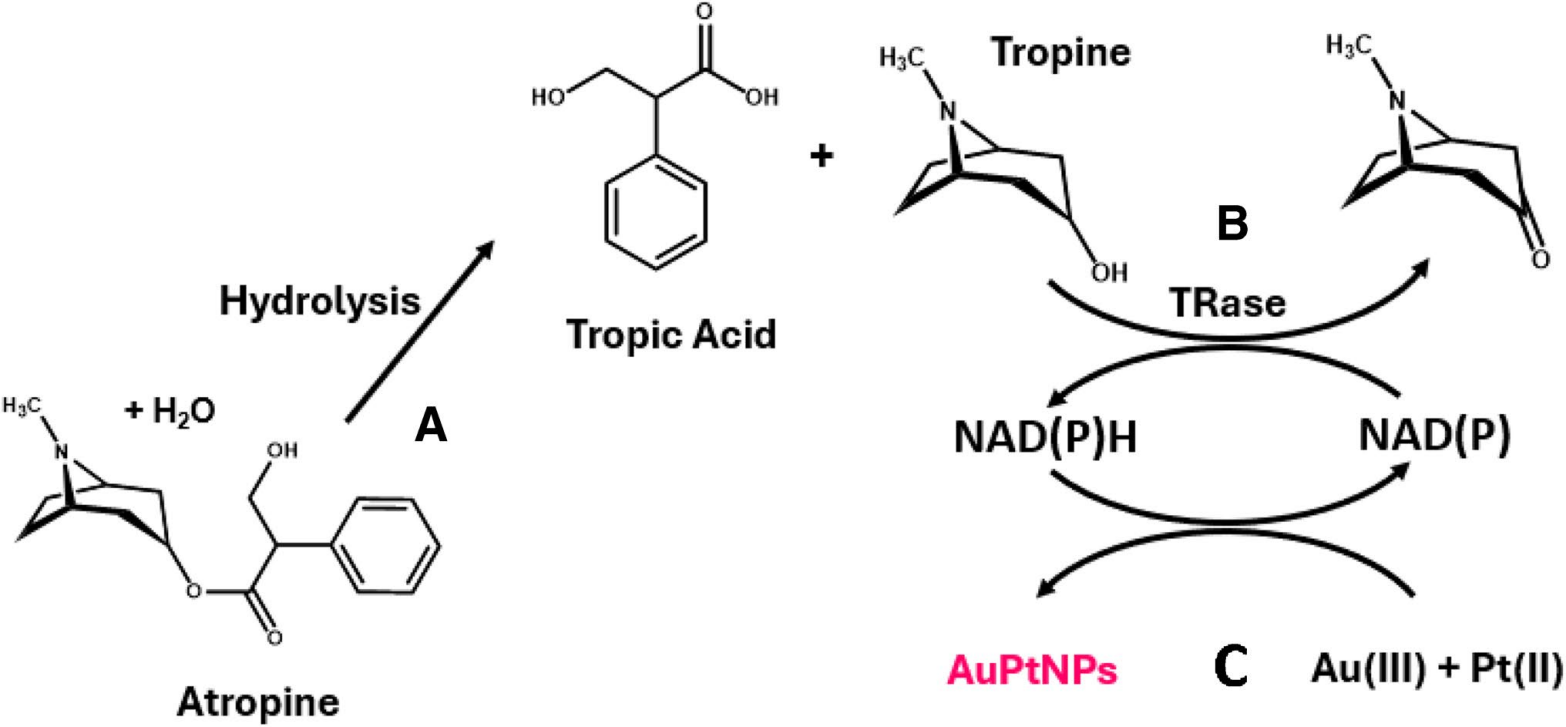
Scheme of the reactions studied in this work

The enzymatic routes of degradation of atropine are known. From an analytical point of view, one of the most interesting consists in coupling the deesterification (enzymatic [17] or chemical) and later oxidation with tropinone reductase [18, 19] (Fig. 1, reaction A and B). To the best of our knowledge, only one previous paper [20] has reported a study involving the coupling of both reactions with an indicating enzymatic reaction based on an organic dye (Diapharose/INT) has been reported for the colorimetric enzymatic determination of this analyte.

New indicating methods based on the optical properties of nanomaterials are currently emerging. These materials are expected to compete with, or even replace, organic chromophores and fluorophores in the development of in-solution methods, optical sensors [monitoring or disposable systems) and fluorescence imaging [21, 22]. The weakness of nanomaterials is that their optical signal lacks sufficient selectivity for the determination of substances in real samples. The combination of enzymes with nanomaterials is then a response to these limitations, acting through different mechanisms (etching, quenching…) most of which require prior synthesis of the nanomaterial [21–23]. Research in this field is opening up new possibilities for optical enzymatic methods of analysis. This paper aims to make a step forward in this direction. It is based on the in situ generation of metallic nanomaterials during NADPH-dependent enzymatic reactions. Previous work of the research group has shown that oxidase-type enzymes are able to reduce the metallic ion to stable nanomaterials whose optical properties (absorption, fluorescence] can be related to the concentration of the corresponding analyte [24, 25].

The first studies exploring the possibility of using the redox properties of NADH to reduce Au(III) as a basis for analytical methods were carried out by Willner´s group [26]. They found that NADH, through its nicotinamide group, can reduce Au (III) in a two-step process:

1 $$Au\left(III\right)+NADH\leftrightarrow Au\left(I\right)+{NAD}^{+}+{H}^{+}$$

2 $$2Au\left(I\right)+NADH\leftrightarrow 2{Au}^{0}+{NAD}^{+}+{H}^{+}$$

where the second step is catalyzed by the previously added seeds of AuNPs (13 nm). Following this idea, it was possible to couple the previous reaction as an indicator method for the enzymatic reaction of lactate with the enzyme lactate dehydrogenase (LDH), but with a short response range (3–6 mM). Later they improved the sensitivity of the method replacing Au(III) with Cu(II) and regrowing AuNPs with Cu^0^ [27]. This methodology of regrowing seed AuNPs by NAD(P)H reaction with ion metals has been used by other authors [28, 29]. Later, the possibility of generating AuNP for the direct reaction between Au(III) and NAD(P)H without seeds [30] was demonstrated, but as far as we know this has not been implemented as an indicating reaction in analytical methods (enzymatic or not).

In this work, the possibility of using NADPH (and NADH) for the in situ generation of AuNPs in the absence of seeds was investigated, as this cofactor can also act as a stabilising agent. The method was coupled to the enzymatic determination of atropine by combining the enzymatic reactions of atropine (Fig. 1A and B) with reactions (1 and 2) as shown in Fig. 1C. This new method does not reach the limits of detection reported by ELISA but is in line with the advantages of enzymatic methods over immunological ones, as described below, and allows matrix interferences to be suppressed efficiently. Interestingly, the generation of nanomaterial makes it possible to discriminate between NADH and NADPH.

## Materials and methods

### Reagents and solutions

All chemicals were used without further purification: Na_2_HPO_4_ ≥ 99% (Panreac 131,679.1211), Na_2_CO_3_ ≥ 99.5% (Sigma)(EC 207–838-8), CH_3_-COONa ~ 100% (VWR Chemicals 27,648.294), tetrachloroauric (III) acid hydrate 99.995% (HAuCl_4_·3H_2_O, Sigma-Aldrich, EC 240–948-4), potassium tetrachloroplatinate (II) 98% (Sigma-Aldrich), β-Nicotinamide Adenine Dinucleotide hydrate NAD (Sigma-Aldrich N1511), Tropine (Sigma-Aldrich 93,550), Tropinone Reductase (TRase) (Gecco Biotech, EC 1.1.1.206).

### Equipment

A Tecnai F30H-7650 microscope (scanning and transmission mode, STEM and high-resolution mode, HRTEM) (FEI, The Netherlands, https://www.fei.com) was used to characterize the gold nanoparticles. Spectroscopic measurements were performed using an Agilent 8453A photodiode UV–vis spectrophotometer, a SPECORD® 210 Plus UV–vis molecular absorption spectrophotometer (one cm cuvettes were used), and a Cary Eclipse fluorescence spectrophotometer (Agilent Technologies (Santa Clara, CA, USA)) equipped with a 96-well microplate reader accessory. The Millipore MiliQ H_2_O system was used for water purification. The temperature of the reactions was controlled by a thermostatic bath connected to the cuvette compartment.

### Deesterification of atropine

A previous study [20] showed that atropine is better deesterified to tropine by hydrolysis in alkaline medium. To do that 20 μL of atropine of the proper concentration was mixed with 20 μL of NaOH 2 M and let to react for 5 min.

### Absorbance measurements in cuvette

Absorbance measurements were carried out in a UV–vis molecular absorption spectrophotometer. PMMA cuvettes with a path length of 1 cm and a final volume of 2 mL were used. Measurements were performed in spectral scan mode, measuring the spectrum from 300 to 800 nm, with measurements taken every 30 s.

For the determination of NADPH, 20 μL of the corresponding NADPH solution was mixed with 1940 μL phosphate buffer pH 7, 20 μL Pt(II) and 20 μL Au(III), the concentration of the metals being the appropriate one for each experiment, and the absorbance was monitored. If the determination was carried out with gold only, the 20 μL of Pt(II) was replaced by 20 μL of phosphate buffer solution.

For the determination of atropine, 20 μL of the corresponding deesterified atropine solution was mixed with 200 μL carbonate buffer pH 10, 40 μL NADP 5 × 10^–3^ M (in aqueous solution), 5 μL TRase 8.8 mg/ml (in buffer pH 7.5) and allowed to react for 5 min. Then, 1675 μL of phosphate buffer pH 7, 48 μL Pt(II) 5 × 10^–2^ M and 12 μL Au(III) 5 × 10^–2^ M were added and the absorbance was monitored.

### Absorbance measurements in 96-well plates by reflectance

When many measurements are required (mainly for systematic optimisation), it is more convenient to use a well plate reader to obtain all the measurements simultaneously. As we do not have access to a specific instrument, the measurements were carried out with a spectrofluorometer according to the following procedure: 1) the fluorescence intensity was acquired with the synchronous scanning function (maintaining Dl = 0 nm between the two monochromators) in the 400–800 nm range; the spectrum obtained for a blank solution corresponded to the *I*_0,*λ*_ value of each wavelength; 2) after the addition of the corresponding solution, the synchronous scanning was performed (Dl = 0 nm) again; the spectrum obtained corresponded to *I*_*t,λ*_; 3) the complete absorption spectrum was then calculated as:

3 $${Abs}_{\lambda }=-log\left(\frac{{I}_{t,\lambda }}{{I}_{O,\lambda }}\right)$$

For the determination of NAD(P)H, 240 μL of phosphate buffer solution pH 7 was mixed with 30 μL of NAD(P)H at the appropriate concentration (in aqueous solution) and 30 μL of a mixture of gold and platinum at the appropriate concentration (in aqueous solution).

### Treatment of the sample: lixiviation from the buckwheat sample

The method developed by Adamse was applied [11]. Four grams of buckwheat is weighed, crushed, and lixiviated in a beaker with 40 mL of a mixture of methanol/water/formic acid in a ratio of 60/40/0.4 (V/V/V). After washing for half an hour, a 4-mL aliquot of the supernatant is filtered by centrifugation through a 10-kDa ultrafilter. The measurement procedure described in 2.4 for atropine was applied to these samples, with 20 μL of atropine solution replaced by 20 μL of sample solution.

## Results

### NADPH determination by nanomaterials generation

**Fig. 2:**
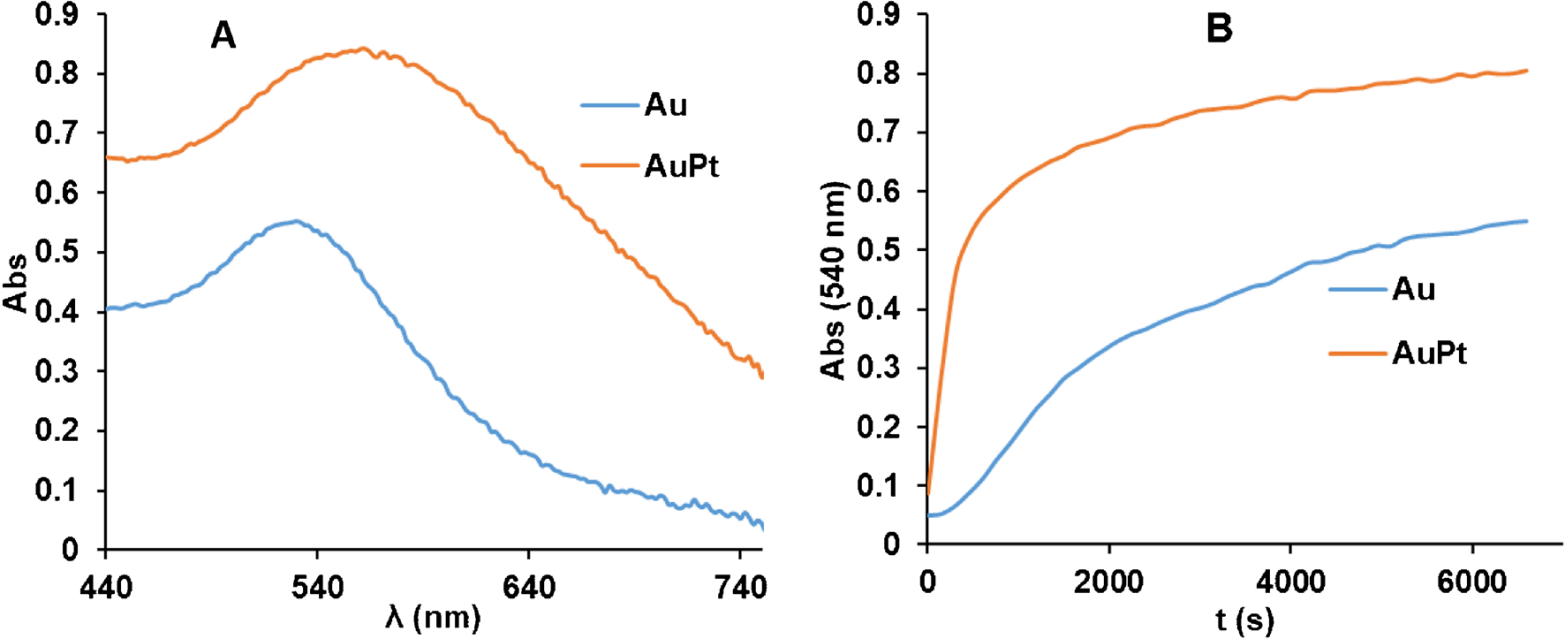
**A**) Molecular absorption spectra of AuNPs and AuPtNPs synthetised in the presence of NADPH and Au/III) or Au(III)/Pt(II), respectively. **B**) Variation with time at Abs_540nm_ of the corresponding nanoparticles. In both cases, a concentration of NADPH of 1 × 10^–4^ M was used

**Fig. 3:**
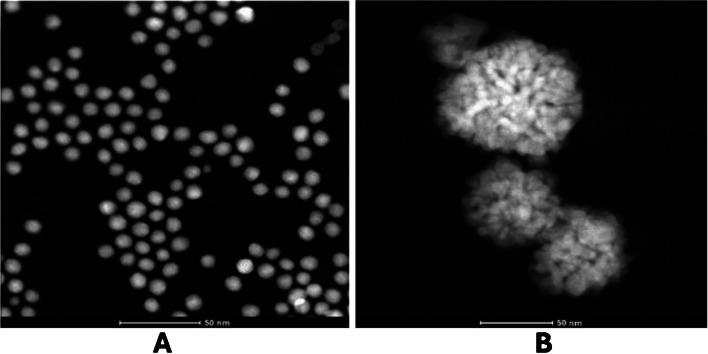
**A**) TEM image of the AuNPs. **B**) TEM image of the AuPtNPs

Our results confirm that NADPH can generate AuNP without seeds with a maximum absorbance at 540 nm. Figure 2 shows the molecular absorption spectra (A) and the absorbance at the maximum (Abs_max_) versus time profile (Abs = f(t)) (B) obtained along the reaction (measurement in cuvette). An S-shaped line is obtained during the first few minutes, which is later distorted to give an increasing signal. Figure 3A shows that spherical and reproducible AuNPs are formed, with a slight tendency to aggregate. During the optimisation study it was observed that the formation of AuNPs from Au (III) and NADPH is highly dependent on the nature of the buffer, the pH, the ionic stretch of the medium and the Au(III) concentration.

The effect of pH is important in all reactions involving NADPH, including enzymatic reactions, and has therefore been studied in detail (Figure S1A). There are two opposing effects. On the one hand, NADPH can degrade over time [31] (following a mechanism explained by Alivisatos [32]), such that the lower the pH of the solution, the faster the degradation [33]. This was confirmed in Figure S1B which shows that the kinetics of NADPH degradation at pH = 5, both in acetic/acetate buffer and in phosphate solution medium, follow a first-order kinetics with constants of the order of 0.1 min^−1^ M^−1^. These results agree with those obtained by We [33]. On the other hand, the normal reduction potential of NADPH increases with increasing pH, so that its reducing power decreases. The optimum pH range found in this work is between 5 and 7. At higher pH, nanoparticles are formed, but more slowly and in smaller proportions.

The buffer concentration is also important. As the buffer concentration increases, so does the degradation effect increase (see the value of the apparent constant, Figure S1B), and also the ionic strength, which has a very important effect on nanoparticle formation (the higher the ionic strength, the higher the absorbance). However, at buffer concentrations above 0.1 M, a second maximum appears at 700 nm (see Figure S1C) with a decrease in absorbance at 540 nm, which may be due to AuNP aggregation reducing the sensitivity at 560 nm. Integrated absorbance over the entire spectral range could be used, but this does not improve sensitivity.

The effect was studied of Au(III) concentration on the analytical figures of merit was studied at the optimum pH and buffer concentration. Figure S2A shows the results obtained. As can be seen, there is a sigmoidal relationship between absorbance and NADPH concentration. Since the lower the Au(III) concentration, the higher the sensitivity and the shorter the response range, the Au(III) concentration can be chosen appropriately depending on the NADPH concentration range to be investigated. Balancing both aspects, a concentration of 1.0 × 10^–4^ M Au(III) was found to be optimal; using this concentration (Figure S2C), the NADPH response range goes from 8.0 × 10^–5^ to 1.7 × 10^–4^ M (see Figure S2B for Abs = f(t) profiles). Both the S-shaped calibration line and the short range have also been obtained by other authors using AuNP seeds [16]. In addition to the response range, the analytical figures of merit of this method are a limit of detection (LoD) of 2.4 × 10^–5^ M, a limit of quantification (LoQ) of 8.0 × 10^–5^ M and a relative standard deviation of 3.4% (1.5 × 10^–4^ M NADPH, *n* = 5). From an analytical point of view, a linear relationship leads to less uncertainty (due to calibration) than an S-shape. Figure S2D shows how Figure S2C can be linearized if desired.

**Fig. 4:**
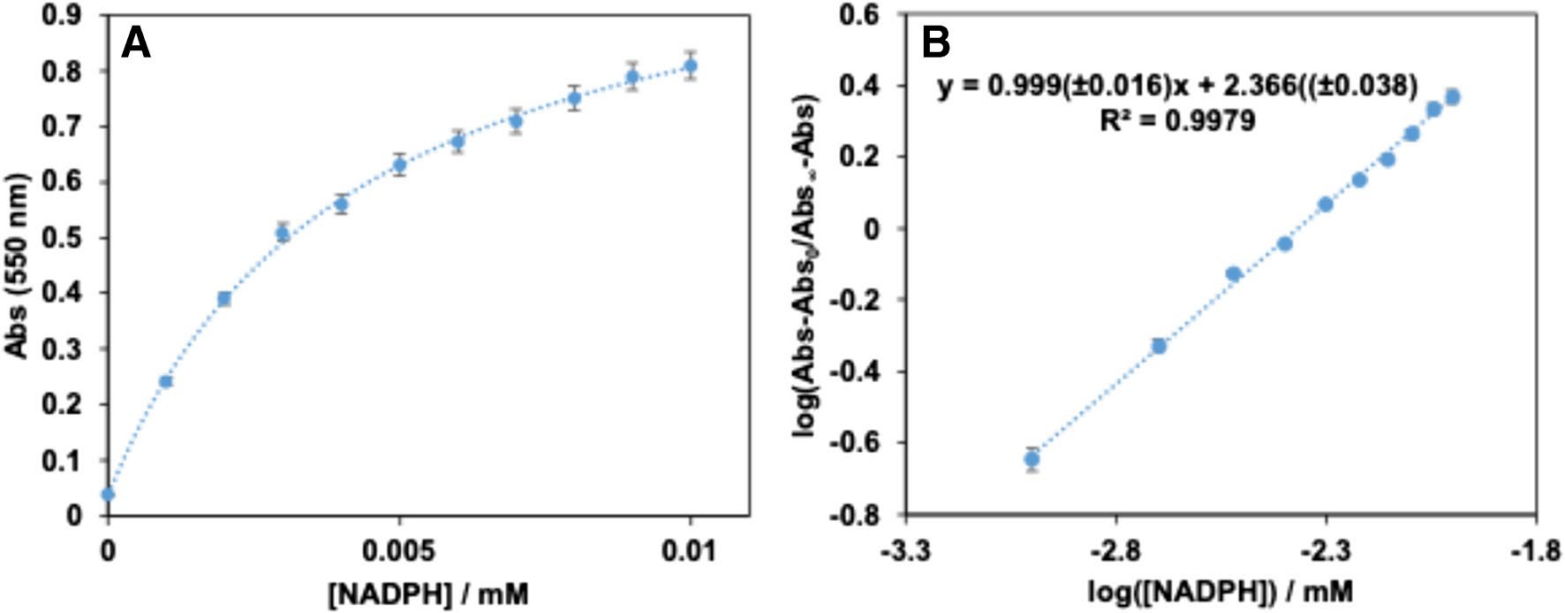
Calibration curve for the reaction using the following concentrations of metals: [Au(III)] = 1 × 10^–4^ M; Pt(II) = 4 × 10^–4^ M. **A**) Abs = f([NADH] representation; **B**) fitting to Eq. (4)

The analytical figures of merit obtained for the in situ generation of nanoparticles from Au(III) during enzymatic reactions can be improved by using Au(III)/Pt(II) mixtures as ion metal precursors [24]. There are two main reasons for this: 1) Pt(II) is able to reduce Au(III) to Au(I); 2) the molar absorptivity of AuPtNP is higher [24, 34], probably due to its nanodendritic shape. This was also tested in this case (Fig. 3B shows the TEM image of the obtained AuPtNPs). First, the Pt(II)/Au(III) ratio was optimized, with the best results obtained at platinum concentrations four times higher than those of gold (Figure S3A). In this case, the addition of Pt(II) together with Au(III) resulted in a color change and a broader absorption spectrum, apart from a different Abs = f(t) representation (Fig. 2). Furthermore, the addition of platinum to the reaction allows the determination of smaller amounts of NADPH as well as having a longer response range. As in the previous section, and also using 0.1 M phosphate buffer solution at pH = 7, with a concentration of Au(III) = 1 × 10^–4^ M together with Pt(II) = 4 × 10^–4^ M, a representation of the absorbance obtained at the absorption maximum (550 nm in this case) was made (Fig. 4A). This representation covers a range of concentrations from 2.4 × 10^–7^ M to 1 × 0·10^–5^ M (Abs = f(t) are shown in Figure S3B). In addition to the response range, the analytical figures of merit of this method were calculated, being 1.0 × 10^−7^ M LoD, 2.4 × 10^–7^ M LoQ and a relative standard deviation of 3.7% (8.0 × 10^–5^ M NADPH, *n* = 5). This S-shaped curve can also be linearized (Fig. 4B) according to Eq. (4). Abs_∞_ and Abs_0_ are the maximum and minimum absorbances, respectively, C_1/2_ is the concentration at half height (inflection point) and B is a dimensionless parameter called the shape factor, which is related to the curvature.

4 $$log\left(\frac{Abs-Abs_0}{{\mathrm{Abs}}_\infty-Abs}\right)=B\;\log\;C-B\;log\;C_{1/2}$$

As can be seen, the use of AuPtNP allows both the determination of lower NADPH concentrations and a wider response range.

The reason why NADPH leads to a higher formation rate of AuPtNP than AuNP was investigated using a mathematical model previously developed by our group based on a classical three-step kinetic model of nanomaterial formation (nucleation, growth and aggregation). This is described in detail in section S2 of the Supplementary Material. Broadly speaking, the nucleation process from Au(III) is slower than the growth process, whereas in the case of starting from Au(III)/Pt(II) both processes are fast and occur simultaneously. Furthermore, in this section S2, it can be observed that the application of the model to the results allows us to obtain the values of the rate constants of each process and shows that the molar absorptivity of AuPtNP is of the order of about 35 times higher than that of AuNP, which justifies the higher sensitivity.

### Differences between NADH and NADPH

**Fig. 5:**
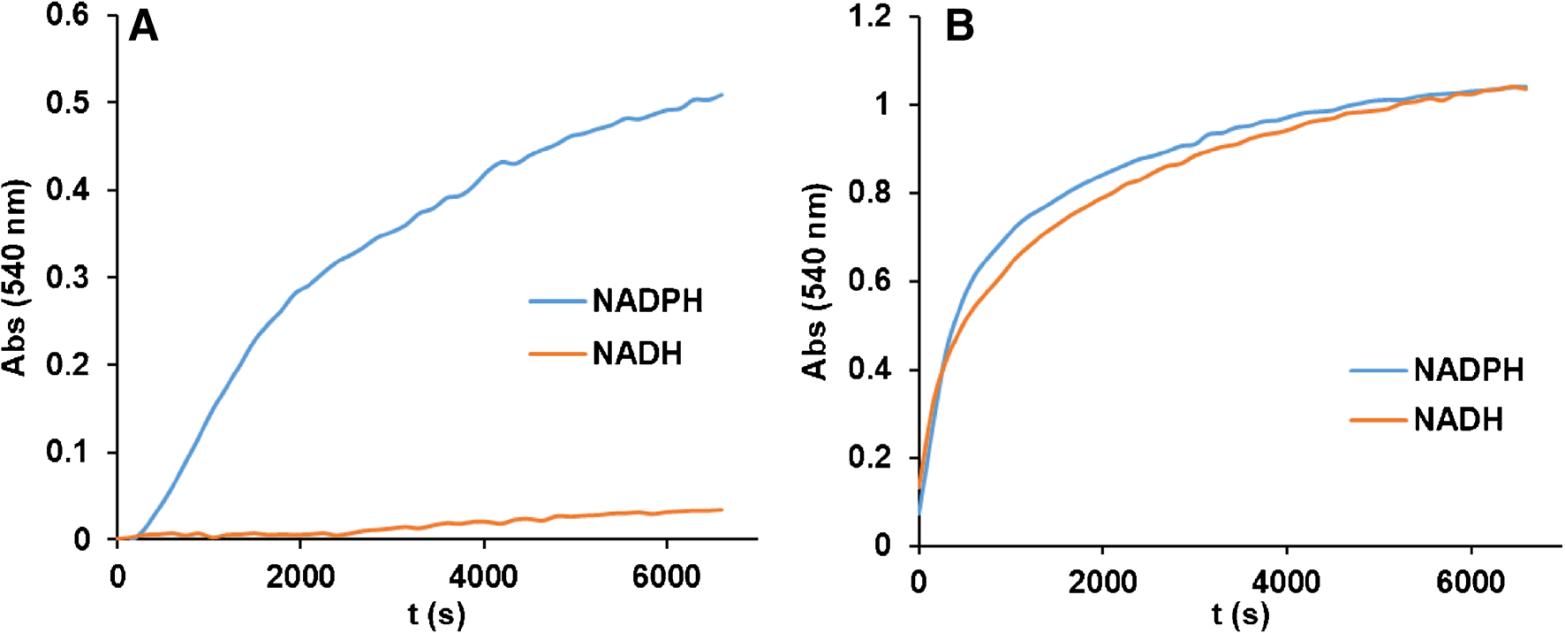
Comparison of the kinetics of the reaction between NADPH and NADH (1,5 × 10^–4^ M for both compounds in A; 1.5 × 10^–5^ M for both compounds in B). **A)** [Au(III)] = 1 × 10^–4^ M.; **B)** [Au(III)] = 1 × 10^–4^ M. [Pt(II)] = 4 × 10^–4^ M

It is well-known that the cofactor of NAD-dependent enzymes can be either NADP or NAD. Many enzymatic reactions can use both, but some others require the specific use of one or the other. For example, glucose-6-phosphate dehydrogenases prefer NADP but others prefer NAD [35, 36]. In addition, the two compounds have different signaling applications in the organism. It may therefore be of interest to distinguish between them. To test this, the generation of AuNPs or AuPtNP from NADP or NAD was investigated. The results are shown in Fig. 5. As can be seen, Au(III) is able to discriminate between the two compounds, since in the same time interval NADH gives practically no AuNP, whereas NADPH does so. However, when Au(III)/Pt(II) is used, both compounds give the same signal (spectra are shown in Figure S5A).

To explain the behavior with Au(III), it is important to consider that NAD and NADP (and their reduced forms) can adopt different conformational structures, namely folded (the most stable in solution) or extended (the most stable when bound to enzymes) (see Figure S5B) [37, 38] and depending on this, the ability to bind to metal ions can change. Published studies have shown that although there are six possible binding sites for metal ion coordination to these dinucleotides, the phosphate and the ribose groups are preferred. However, the final results are not in complete agreement as to which of the two sites is most likely.

For NAD, studies carried out by Hoffman et al. [37] with Cu(II) showed that the complex responds to a 2:1 NAD:Cu(II) stoichiometry, with the Cu(II) coordinating to the ribose of the adenosine group of each NAD (Figure S5C). However, Herrero et al. [39] suggest coordination via the phosphate group involving adenosine. For NADP, the results obtained by Green et al. [40] with Mn(II) and Mondelli et al. [41] with Mg(II) and Cr(III) indicate that the metal ion is bound to the three phosphates (Figure S5D). Recent studies by Kruszynski et al. [42] with NADPH and LiCoO_2_ confirm this and show that binding to the phosphate-ribose moiety is essential for the metal ion reduction. Furthermore, externally added phosphate (i.e., as a buffer) inhibits the interaction of the ribose with the metal ion [42]. It can therefore be concluded that the different behavior of NAD and NADP with Au(III) is due to the different way in which the metal ion coordinates the dinucleotide.

Pt(II) is able to reduce Au(III) to Au(I), so the complexes are formed on this species. There are not many studies on monovalent metal ions. Those published for Li(I) with NAD (by X-ray crystallography) [43] show that it is bound to two NAD molecules. If Au(I) follows this behavior, the same bond is formed whether it is a molecule of NADH or NADPH and no differences are observed between them.

These results show and justify that these reactions can be used both for the joint determination of NADH and NADPH and for the differentiation of the two molecules. For a joint determination, the total concentration (NADH + NADPH) would first be determined using the reaction in the presence of platinum, since both analytes have the same sensitivity in their linear range. The concentration of NADPH would then be determined using the reaction without platinum, as NADH does not react in this case.

### Coupling to the tropinone reductase reaction: application to real samples

As mentioned above, the aim of the work was to couple the formation of AuNPs to the enzymatic reaction of atropine with tropinone reductase (TRase). During sample preparation, atropine has already been hydrolysed to tropine, so the analysis is actually performed on tropine, skipping the previous step. The optimum conditions for tropine oxidation using reaction of Fig. 1B have already been studied and are pH = 10 (carbonare buffer) and 0.022 mg/mL TRase concentration. Comparing these experimental conditions with those obtained for the generation of nanomaterials from NADPH, the pH must be changed between tropine oxidation (pH = 10) and nanoparticle formation (pH = 7), since it is not possible to find a compromise pH for carrying out both reactions simultaneously.

Therefore, the oxidation reaction is first carried out in low volume at pH 10, using the optimised NADP concentration (1 × 10^–4^ M), and then brought to pH 7 with the addition of Au(III) or Au(III)/Pt(II). Very small signals due to nanoparticles were observed with Au(III) alone, but large signals were observed with Au(III)/Pt(II) (Fig S6A).

The main parameters to be optimised were the concentrations of Au(III) and Pt(II). Always maintaining a ratio of 1:4 (Au:Pt) between them, tests were carried out with different gold concentrations (Figures S6B, S6C), finally selecting 4 × 10^–4^ M of Au(III) (and, respectively, 1 × 6·10^–3^ M of Pt (II)) as the optimum concentration.

From the analytical point of view, the most important difference between AuPtNP generation when NADPH is added directly or generated during the TRase reaction with tropine is the nature of the Abs = f(t) profiles obtained. As can be seen in Figure S7, the kinetics of AuPtNP generation depends on the atropine concentration used, but all the profiles tend to the same final absorbance. In this case the analytical parameter that can be related to the atropine concentration could be either the absorbance obtained at a given time or the area of the Abs = f(t) profile at a chosen time. Both parameters give good results, but better precision was obtained using the area.

**Fig. 6:**
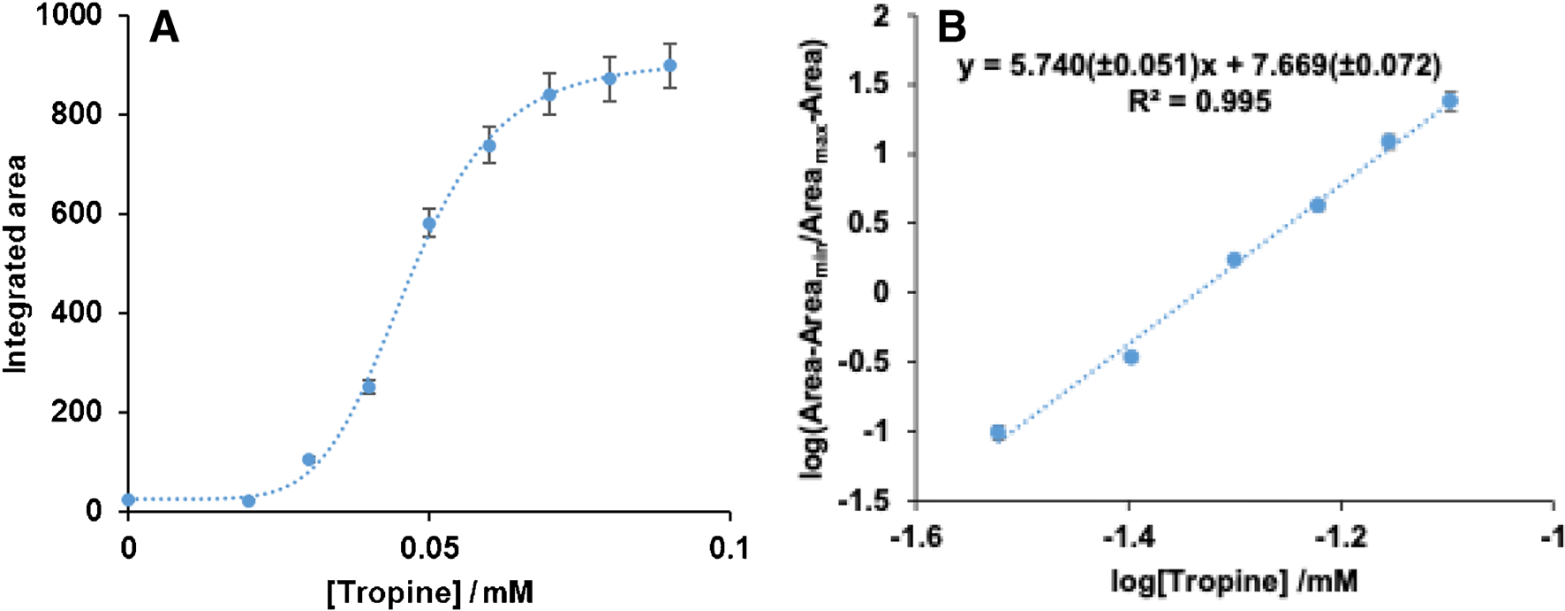
Calibration lines obtained for atropine determination with TRase, Au(III) and Pt(II). **A**) Calibration line; **B**) logistic curve for the reaction using the following concentrations of metals: [Au(III)] = 4 × 10^–4^ M; Pt(II) = 1 × 6·10^–3^ M

Figure 6A shows the integrated area of Abs = f(t) profiles obtained at 540 nm for each atropine concentration from 0 to 2000s. An S-shaped curve is obtained which, after applying the transformation given by (4), again gives a linear relationship (Fig. 6B). Each measurement is the average of three different values. The relative standard deviation obtained was 3% (8 × 0·10^–5^ atropine, *n* = 5), and the LoD and LoQ were 0.0204 mM and 0.0253 mM, respectively. The LoD and LoQ were calculated using the calibration curve where the area to be interpolated would be the value obtained by calculating: Area_b_ = 3s_b_ + Area_min_ and Area_b_ = 10s_b_ + Area_min_, respectively. The linear range goes from 0.025 to 0.09 mM.

The sensitivity obtained is slightly lower than that of enzymatic methods based on the classical colorimetric or fluorometric properties of a dye. Nonetheless, it has several advantages, for example: additional enzymes are not required, many of the lateral reactions that HRP can suffer (such as the reaction with phenols) or the oxidized form of the dye are avoided, the oxidation of the dye is prevented, and interferences caused by H_2_O_2_ reactive species are also supressed.

The method was then applied to the determination of atropine in buckwheat samples. A commercial buckwheat was first subjected to the sample treatment described in Sect. 2.5. As the sample gave an atropine concentration below the limit of quantification of the method, it was fortified with a constant atropine concentration (6 × 10^–5^ M). Different volumes of matrix were subjected to the described procedure, but recoveries lower than 100% were obtained (Table S1), revealing proportional interferences. Although the calibration line obtained fits a 4-parameter logistic curve, the standard addition method can still be applied. The method developed is described in the Supplementary Material (Annex 1); most importantly, the C_1/2_ and B values are not affected by matrix interferences The procedure is as follows:

1. Prepare a sample solution and another sample solution with a high concentration of the spiked analyte (such that its concentration corresponds to the upper flat zone of Fig. 6A). The absorbance values of both solutions are measured (Abs_s_ and Abs_∞,s_, respectively).
2. The Abs_∞,s_ is divided by the Abs_∞_ value of the calibration line to obtain a correction factor (P). The Abs_0_ value is then multiplied by P to obtain Abs_0,s_. The value of P depends on the sample to be analysed and on the sample preparation procedure (sample weight, sample volume, solvent volume, and volume taken for the determination).
3. The values of Abs_s_, Abs_∞,s_, and Abs_0,s_ are substituted in the equation of the calibration line and the concentration value is obtained.

To test this procedure, the sample was spiked with four different concentrations of atropine. The results obtained (Table S2) showed an average recovery of 96.9 ± 2.0% (*n* = 4).

## Conclusions

This paper demonstrates that the formation of gold nanostructures by reaction with NADPH formed after enzymatic reactions involving dehydrogenase enzymes is feasible, does not require nanoparticle seeds and can be applied to the determination of atropine in buckwheat samples. This method avoids the use of indicator reactions involving chemical dyes and peroxidase. Although a reasonably good sensitivity is obtained, it is slightly lower than the dyes/peroxidase systems, and improvements need to be made, probably involving the in situ generation of other nanomaterials. In addition, the application of this method to other substrates and other dehydrogenase-type enzymes needs to be investigated. Furthermore, the generation of AuNP from Au(III) allows us to differentiate between NADH and NADPH, opening up new opportunities for simultaneous enzymatic determinations or selective studies of these dinucleotides.

## Supplementary Information

Below is the link to the electronic supplementary material.

Supplementary file1 (PDF 921 KB)

## Data Availability

No datasets were generated or analysed during the current study.
