# Supplementary material for: NADPH-mediated seedless in situ formation of gold or gold-platinum nanoparticles for the enzymatic determination of atropine
Source: Mikrochim Acta. 2025 Feb 4;192(3):132. doi: 10.1007/s00604-025-06964-x (PMC11794361; doi:10.1007/s00604-025-06964-x)
Supplement: Supplementary file 1 — Supplementary file1 (PDF 921 KB) [file 604_2025_6964_MOESM1_ESM.pdf]

Supplementary Material

NADPH-mediated seedless in-situ formation of gold or gold-platinum nanoparticles for the enzymatic determination of atropine

M. Domínguez, S. de Marcos, J. Galbán

Analytical Chemistry Department, University of Zaragoza, Zaragoza 50009, Spain.
Instituto de Nanociencia y Materiales de Aragón (INMA), CSIC-Universidad de Zaragoza, Zaragoza 50009, Spain

SUMMARY

Section S1.- NADPH determination **2**
Figure S1A. pH and buffer effect ………………………………………………….. 2
Figure S1B. Stability in acetate and phosphate (pH=5) ………………………..……. 2
Figure S1C. Optimization of ionic strength effect ………………..……………..….. 3
Figure S2A. Effect of the Au(III) concentration …………………………….……….. 3
Figure S2B. Abs_{AuNP}=f(t) for different NADPH concentrations…………………….…. 3
Figure S2C. Calibration line obtained for NADPH: optimum [Au(III)]……………..…. 4
Figure S2D. S-shaped curve linearization ………………………………………... 4
Figure S3A. Effect of Pt(II) concentration on final absorbance ……….……..….. 5
Figure S3B. Abs=f(t) profiles of the calibration line for AuPtNP ……………….…. 5

Section S2.- Kinetic study of AuNP and AuPtNP formation from NADPH **5**
Figure S4A. Experimental and theoretical Abs=f(t) profiles (AuNP) ……………… 6
Figure S4B. Experimental and theoretical Abs=f(t) profiles (AuPtNP) …………… 7

Section S3.- Differences between NADPH and NADH **8**
Figure S5A. UV-spectra of nanoparticles obtained from NADH and NADPH …... 8
Figure S5B. Conformational forms of NAD and NADP ………………………….…. 8
Figure S5C. $Cu(II)(NAD)_2$ complex ……………………………………………….. 8
Figure S5D. Mn(II)(NADP) complex ………………………………………………. 9

Section S4.- Formation of AuPtNPs: Atropine determination **9**
Figure S6A. Molecular absorption spectra of the formed AuNPs or AuPtNPs ….. 9
Figure S6B. Optimization of metal ions in atropine determination: spectra……… 9
Figure S6C. Optimization of metal ions in atropine determination: Abs=f(t)…….. 10
Figure S7. Abs=f(t) profiles obtained during the calibration study (Atropine)……. 10
Table S1. Application to real samples: Direct sample analysis……………………. 10
Table S2. Application to real samples using the standard addition method…….. 11

Annex 1. The standard addition method in 4-parameter logistic calibration **11**

Section S1.- NADPH determination

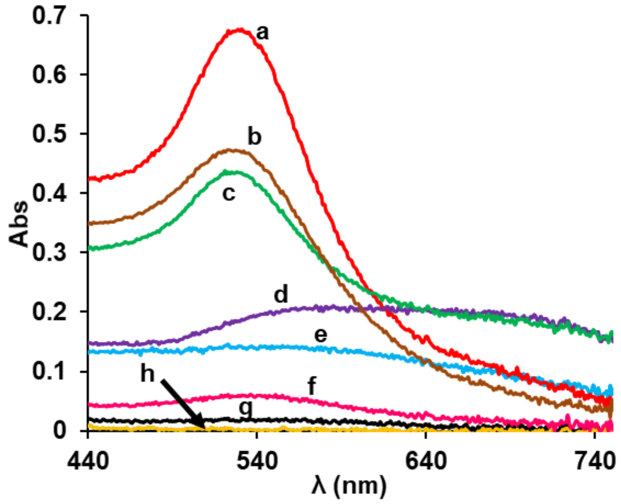

Figure S1A: pH and buffer effect. Optimization of the pH. a) pH 5 with acetate. b) pH 7 with phosphate. c) pH 5 acetate and phosphate. d) pH 11 phosphate. e) pH 3 phosphate. f) pH 9 carbonate and phosphate. g) pH 9 carbonate. h) pH 9 TRIS. In all cases [Au(III)]=1.0×10^{-4}M and [NADPH]=2.0×10^{-4}M

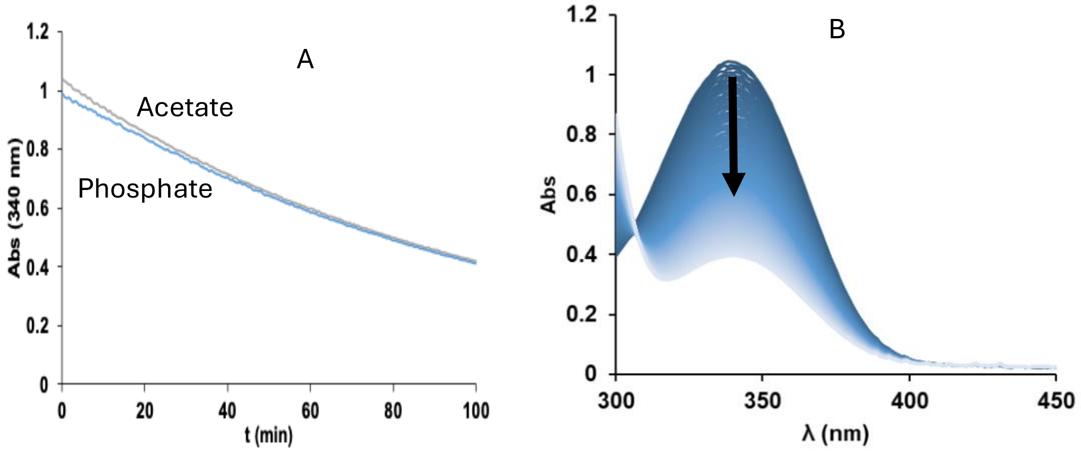

Figure S1B: Stability in acetate and phosphate (pH=5). Instability of NADPH at pH=5. **A)** kinetic profile at 340 nm for acetate and phosphate, **B)** kinetic Spectra (acetate). The data of figure A fit the equations

$$log\frac{Abs_0}{Abs_t} = -k't = -\frac{kC_{buffer}}{2.303}$$

Abs_0 being the Absorbance for t=0, t is time in min, k´ being the pseudo first-order kinetic constant, k being the kinetic constant and C_{buffer} being the buffer concentration used.

For Acetate:

$$log\frac{Abs_0}{Abs_t} = -0.0039t - 0.006 \qquad R^2 = 0.9998$$

$$k' = \frac{k\ C_{acetate}}{2.303} = 0.0039 \ ==> k = 0.090\ min^{-1}M^{-1}$$

For Phosphate:

$$log\frac{Abs_0}{Abs_t} = -0.0038t + 0.003 \qquad R^2 = 0.9998$$

$$k' = \frac{k\ C_{phosphate}}{2.303} = 0.0038 \ ==> k = 0.088\ min^{-1}M^{-1}$$

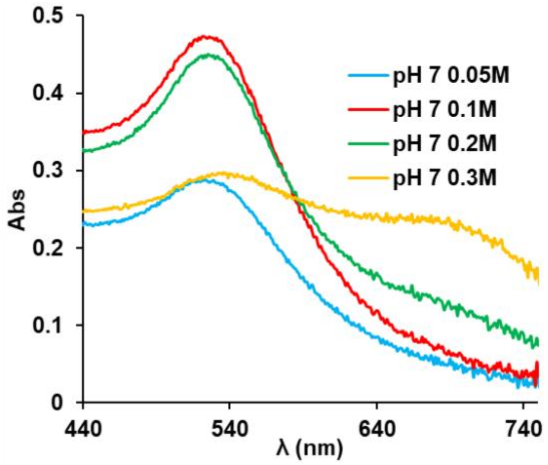

Figure S1C. Optimization of ionic strength. In all cases pH=7 phosphate buffer, [Au(III)] = $1.0x10^{-4}$ M and [NADPH] = $2.0x10^{-4}$M.

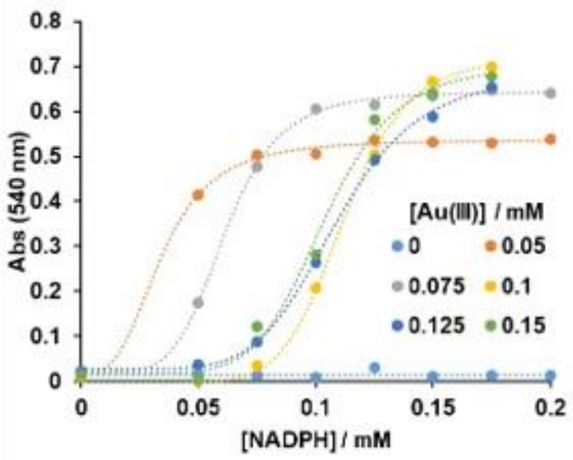

Figure S2A. Effect of the Au(III) concentration in the formation of AuNPs with NADPH. In all cases 0.1M phosphate buffer at pH=7.

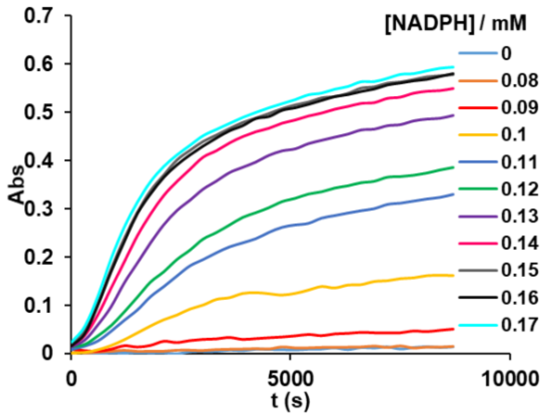

Figure S2B. Abs_{AuNP}=f(t) for different NADPH concentrations. All measurements with [Au(III)] = $1x10^{-4}$ M at pH=0.7 and 0.1M phosphate buffer.

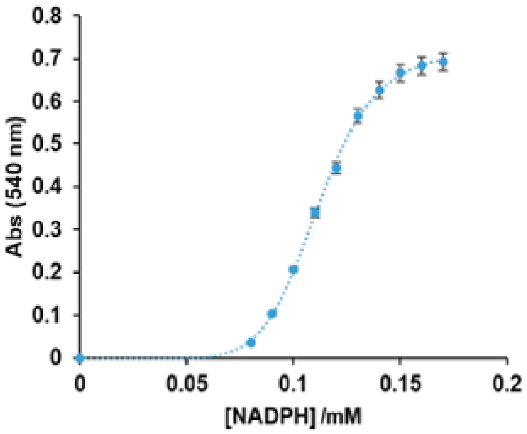

Figure S2C. Calibration line obtained for NADPH. [Au(III)]= $1x10^{-4}$M, 0.1M phosphate buffer at pH=7.

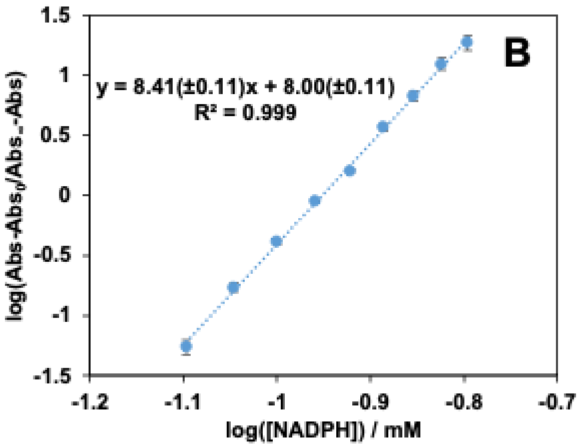

Figure S2D. S-shaped curve linearization.

This figure shows how the S-shaped curve obtained can be linearized. To do that it is important to note that the sigmoid curve can be mathematically fitted to the so-called 4-parameter logistic curve, given by:

$$Abs = Abs_{\infty} + \frac{Abs_0 - Abs_{\infty}}{1 + \left(\frac{C}{C_{1/2}}\right)^B} \qquad (S1)$$

Abs is the absorbance obtained for each of the concentrations (C), Abs_{∞} and Abs_0 are the maximum and minimum absorbances, respectively, $C_{1/2}$ is the concentration at half height (inflection point) and B is a dimensionless parameter called the shape factor, which is related to curvature. Rearranging the equation and taking logarithms gives a mathematical expression that fits a straight line (Fig. 4B) following the equation:

$$log\left(\frac{Abs - Abs_0}{\mathrm{Abs\infty} - Abs}\right) = B \log C - B\ log\ C_{1/2} \qquad (S2)$$

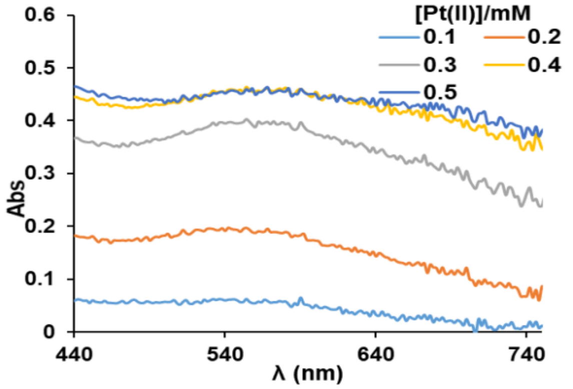

Figure S3A. Effect of Pt(II) concentration on final absorbance. Final spectra obtained (by reflectance) of the AuPtNPs with $1x10^{-5}$ M NADPH, $1x10^{-4}$ M Au(III) and different concentrations of Pt(II).

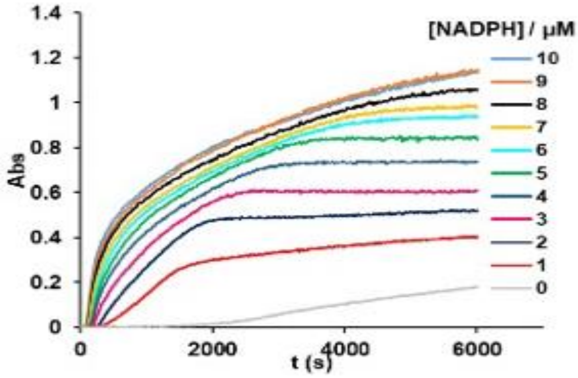

Figure S3B. Abs_{AuPtNP} = f(t) profiles obtained for different NADPH concentrations in the optimal conditions found for in-situ AuPtNP generation. [Au(III)] = $1x10^{-4}$ M. [Pt(II)] = $4x10^{-4}$ M.

Section S2.- Kinetic study of AuNP and AuPtNP formation from NADPH.

A kinetic/mathematical model has been previously developed see references [24,25] of the main manuscript for nanoparticle formation during the analytical reactions consisting of a three-step mechanism:

1.- Nucleation $$M^{n+} + NADPH \rightarrow k_1 \rightarrow M^0 \quad \text{(S3)}$$

2.- Growth $$M^{n+} + NADPH + M^0 \rightarrow k_2 \rightarrow MNP \quad \text{(S4)}$$

3.- Aggregation $$nMNP \rightarrow k_3 \rightarrow MNP_n \quad \text{(S5)}$$

M^{n+} being the concentration of the metal ion precursor (in this case Au(III)) and MNP is the metal nanoparticle (MNP) formed. For the first two steps we developed a mathematical equation based on the Finke-Watzky model[1] . To describe the last step of aggregation, it was decided to use the Avrami equation, which is suitable for explaining

[1] Watzky MA, Finke RG (1997). J Am Chem Soc. 119: 10382-10400. https://doi.org/10.1021/ja9705102

processes of general transformation of a liquid phase into a solid phase. In this case, the aggregation step must be included ($Abs_t = \left(1 - e^{-k_3 t}\right)$), and the final mathematical model obtained is given by:

$$Abs_t = \varepsilon_{AuNP,n}\frac{k_1}{k_{2,n}}\left(\frac{1-e^{-C_{Au}(k_1+k_{2,n}C_{NADPH})t}}{e^{-C_{Au}(k_1+k_{2,n}C_{NADPH})t}+\frac{k_1}{k_{2,n}C_{NADPH}}}\right)\left(1-e^{-k_3 t}\right) \qquad \text{(S6)}$$

being $\varepsilon_{AuNP,n}$ the molar absorptivity per atom, k_1 and k_2 the kinetic constants for the nucleation and growth steps, respectively, and k_3 the Avrami constant.

When this model is applied to the Abs=f(t) representations obtained during the NADPH calibration study (Fig S2B) based on AuNP from Au(III), the following values are obtained: $\varepsilon_{AuNP,n} = 3700 \pm 200\ M^{-1}cm^{-1}$, $k_1 = 15.55 \pm 6.14\ M^{-1}s^{-1}$, $k_2 = 7.86{\cdot}10^4 \pm 2.61{\cdot}10^4\ M^{-2}s^{-1}$ and $k_3 = 3.84{\cdot}10^{-4} \pm 0.95{\cdot}10^{-4}\ M^{-1}s^{-1}$. As can be seen, the nucleation step is slower than the growth step, which may be the reason why some authors have not observed AuNP formation without seeds. Figure S4A shows that the experimental Abs=f(t) profiles agree with those predicted by Eq. (S6) for different NADPH concentrations.

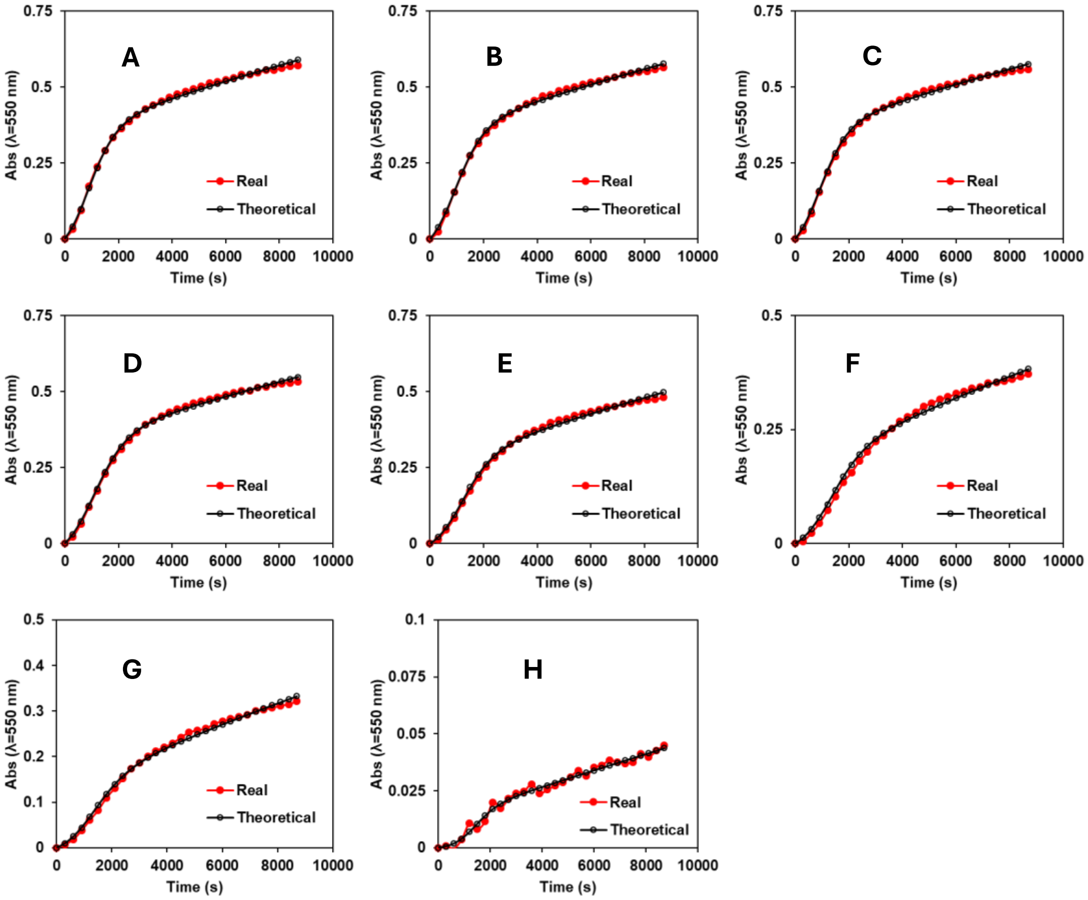

Figure S4A. Experimental and theoretical Abs_{AuNP}=f(t) profiles. Comparison between experimental and theoretical profiles (according to equation S6) corresponding to some of the NADPH concentrations (in mM) in figure S2B: A) 0.17; B) 0.16; C) 0.15; D) 0.14; E) 0.13; F) 0.12; G) 0.11; H) 0.09

However, when the model is applied to the Abs=f(t) profiles obtained for AuPtNP the S-shaped profile is not always shown (fig S3B), indicating that the first two steps (nucleation and growth) cannot be separated (both are included in $k_{1/2}$), so that the full mechanism

consists of two steps: nucleation-growth and aggregation. This type of model has also been described previously by our group [24], and fits to the following equation:

$$Abs_t = \varepsilon_{AuNP,n} c_{NADPH} c_{Au} \left(\frac{e^{(c_{Au}-c_{NADPH})k_{1/2}t} - 1}{c_{Au} e^{(c_{Au}-c_{NADPH})k_{1/2}t} + c_{NADPH}} \right) \left(1 - e^{-k_3 t}\right) \qquad (S7)$$

Figure S4B shows the Abs=f(t) corresponding to the points of the calibration line and the comparison with the theoretical fit of these points according to equation (S7). From the Abs=f(t) representations obtained for the calibration points, the following values are obtained: $\varepsilon_{AuPtNP,n} = 124.45 \cdot 10^3 \pm 0.42 \cdot 10^3$ $M^{-1}cm^{-1}$, $k_{1/2} = 50 \pm 20$ $M^{-1}s^{-1}$ and $k_3 = 2.58 \cdot 10^{-4} \pm 0.46 \cdot 10^{-4}$ $M^{-1}s^{-1}$. As expected, $\varepsilon_{AuPtNP,n}$ is much higher than $\varepsilon_{AuNP,n}$ (about 35 times higher). Regarding $k_{1/2}$, it is difficult to compare with the results in the previous section because the constant includes both nucleation and growth steps. Note that k_3 is similar to that calculated for AuNP, so the aggregation step does not depend on the previous steps.

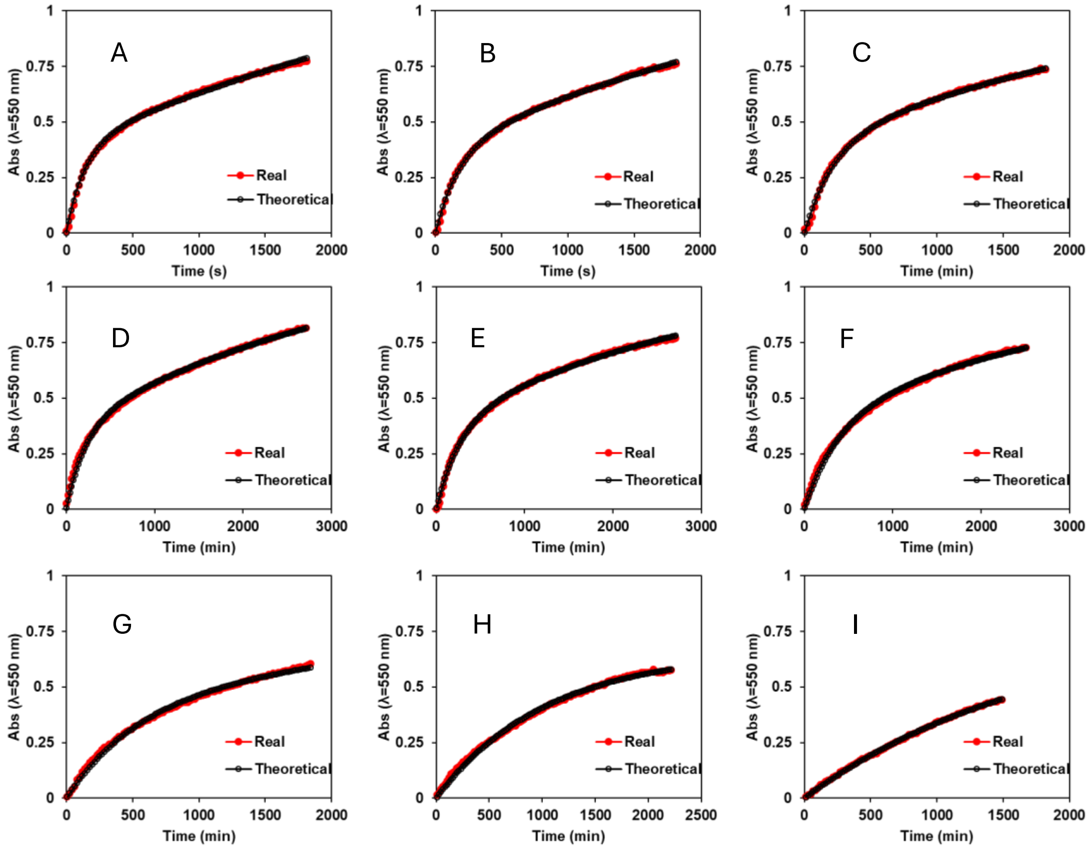

Figure S4B. Experimental and theoretical Abs_{AuPtNP}=f(t) profiles. Comparison between experimental and theoretical profiles (according to equation S7) corresponding to some of the NADPH concentrations (in mM) in figure S3B: A) 10 ; B) 9.0 ; C) 8.0 ; D) 7.0 ; E) 6.0 ;F) 5.0; G) 4.0 ; H) 3.0 ; I) 1.0 .

Section S3.- Differences between NADPH and NADH

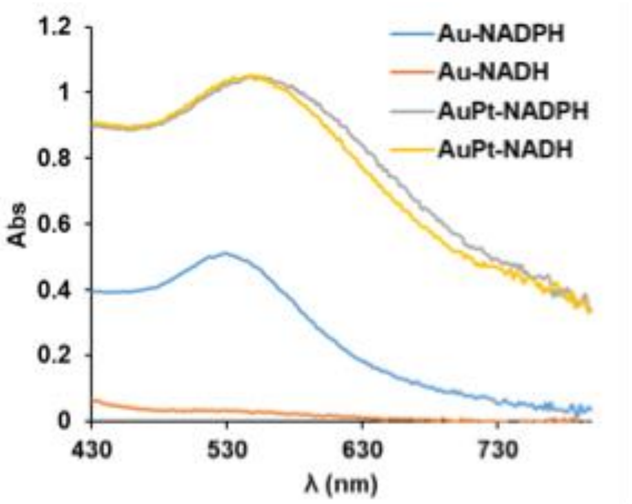

Figure S5A. UV-vis spectra of nanoparticles obtained from NADH and NADPH. Comparison of molecular absorption spectra of nanomaterials obtained from NADH and NADPH using Au(III) or Au(III)/Pt(II). $1.5x10^{-5}$ M NADPH and NADH concentrations. [Pt(II)] = $4x10^{-4}$ M and/or [Au(III)] = $1x10^{-4}$ M

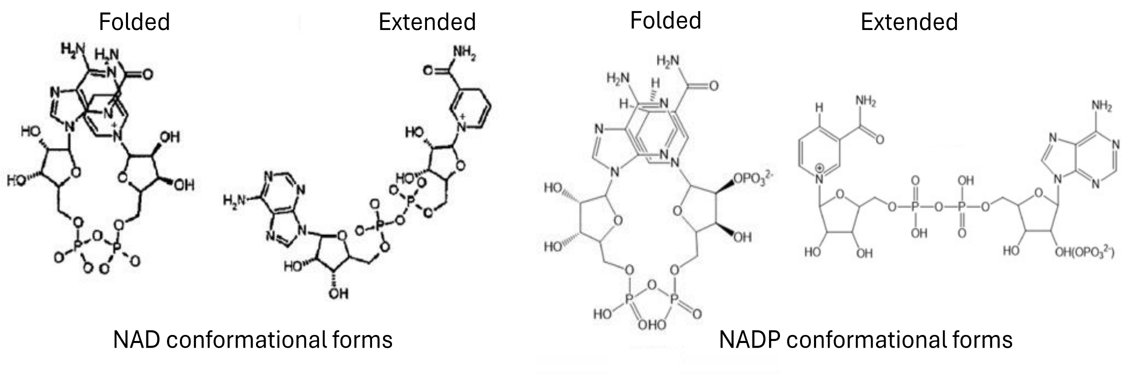

Figure S5B. **Conformational forms of NAD and NADP** according to references [38] and [39] in the main manuscript.

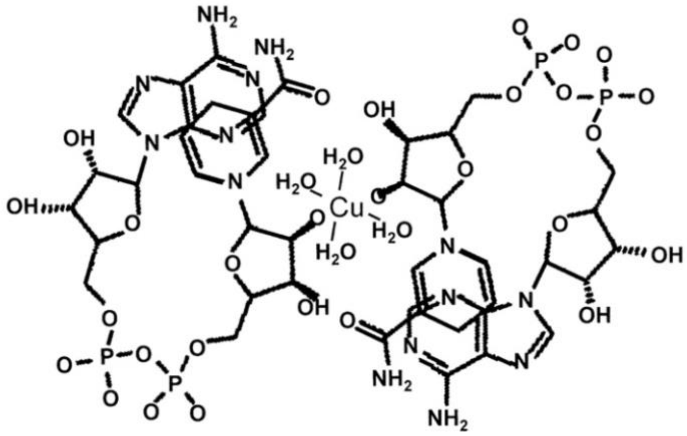

Figure S5C. **Cu(II)(NAD)$_2$ complex** according to reference [38] in the main manuscript

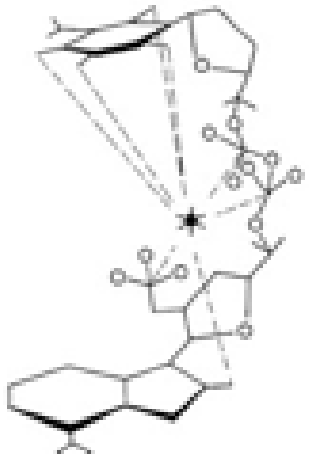

Figure S5D. **Mn(II)(NADP) complex** according to reference [41]

Section S4.- Atropine determination

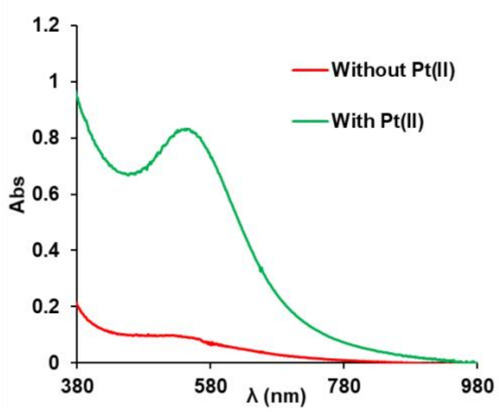

Figure S6A. Molecular absorption spectra of the AuNPs and AuPtNPs nanoparticles formed during the enzymatic reaction of atropine/Tropinone reductase/NAD in the presence of Au(III) and Au(III)/Pt(II), respectively.

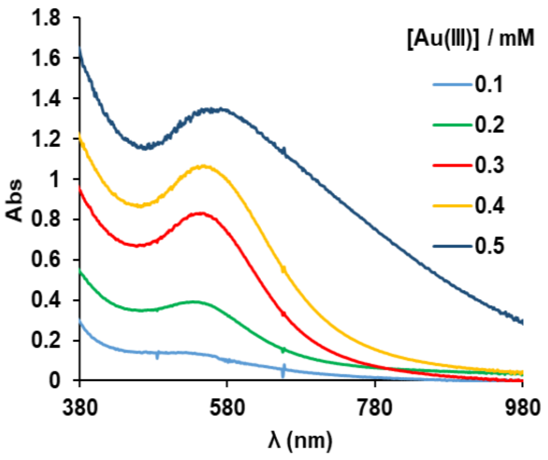

Figure S6B. **Optimization of metal ions in atropine determination: spectra**. Effect of Au(III) concentration on AuPtNPs formation. In all cases the [Pt(II)]:[Au(III)] ratio was kept at 1:4.

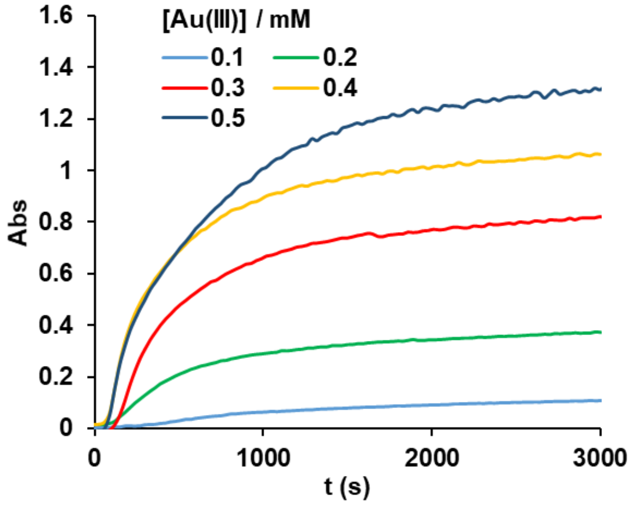

Figure S6C. Optimization of the metal ions in the determination of atropine: Abs=f(t). Kinetics profiles obtained at 550 nm for each Au(III) concentration ([Pt(II)] = 4x[Au(III)] in each case).

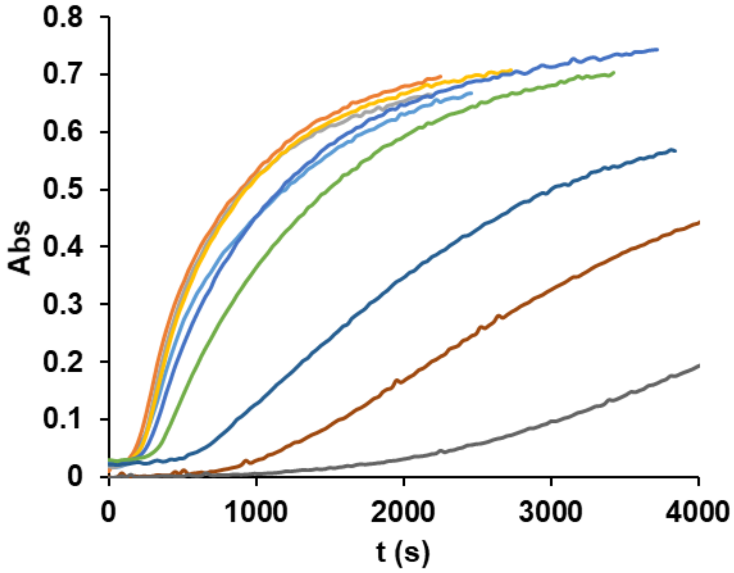

Figure S7. Abs=f(t) profiles obtained during the calibration study (atropine). Kinetic profiles obtained at 550 nm for each atropine concentration used from 2x10^{-5} M to 1x10^{-4} M.

[Atropine] / mM	Integrated area obtained	Integrated area expected	Recovery (%)
0.03	61.21	81.63	74.98
0.04	192.35	273.77	70.26
0.05	417.50	559.39	74.64
0.06	548.94	752.19	72.98
0.08	649.27	876.09	74.11

Table S1. Application to real samples: direct sample analysis. Recoveries obtained after applying the method to spiked samples.

[Atropine]/mM	Integrated area	[Atropine obtained] / mM	Recovery (%)
0.03	58.04	0.029	97.46
0.04	178.65	0.038	95.26
0.05	413.59	0.049	99.41
0.06	525.47	0.057	95.35

Table S2. Application to real samples using the standard addition method. Recoveries obtained for different spiked samples using the standard addition method.

Annex 1. The standard addition method in 4-parameter logistic calibration

It is well known that matrix interferences can be corrected by the standard addition method because they affect each absorbance value in the same proportion.

Our method follows the 4-parameter logistic curve given by equation (S1). If the analyte in the sample is affected by proportional interferences, the new calibration line is:

$$Abs_s = Abs_{\infty,s} + \frac{Abs_{0,s} - Abs_{\infty,s}}{1 + \left(\frac{C}{C_{1/2}}\right)^B} \qquad (S8)$$

where the new absorbances (represented by the subindex "s") are related to the original absorbances (Abs) by a proportionality factor (P):

$$Abs_s = P * Abs \qquad (S9)$$

When S9 is used in equation S8:

$$Abs * P = Abs_\infty * P + \frac{Abs_0 * P - Abs_\infty * P}{1 + \left(\frac{C}{C_{\frac{1}{2}}}\right)^B} ==> Abs = Abs_\infty + \frac{Abs_0 - Abs_\infty}{1 + \left(\frac{C}{C_{\frac{1}{2}}}\right)^B} \qquad (S10)$$

This means that the $C_{1/2}$ and B values of the calibration line are not affected by the proportional interferences. This is clearer when the logarithmic equation is used:

$$log\left(\frac{Abs - Abs_0}{\mathrm{Abs}\infty - Abs}\right) = B\log C - B\ log\ C_{1/2} \qquad (S11)$$

In the presence of proportional interferences:

$$log\left(\frac{Abs_s - Abs_{0,s}}{Abs_{\infty,s} - Abs_s}\right) = log\left(\frac{Abs * P - Abs_0 * P}{Abs_\infty * P - Abs * P}\right) = log\left(\frac{Abs - Abs_0}{Abs_\infty - Abs}\right) \ (S12)$$

which indicates that the logarithmic parameter is not affected by proportional interferences.

Therefore, the methodology for correcting for proportional interferences in this type of calibration is as follows:

1.- Calculate the parameter P. To do this, prepare a sample containing a concentration of spiked analyte such that the signal obtained belongs to the upper flat part of the calibration line; the absorbance of this solution is $Abs_{\infty,PI}$. The P value is obtained by dividing this by the Abs_{∞} of the calibration line.

2.- With P, the $Abs_{0,s}$ is calculated from the Abs_0 of the calibration line:

$$Abs_{0,s} = P * Abs_0$$

3.- Replace $Abs_{0,s}$, Abs_s and $Abs_{\infty,s}$ in equation S8 or S12, where the $C_{1/2}$ and B values are those of the calibration line.
